# Geometric Non-Linear Analysis of Auxetic Hybrid Laminated Beams Containing CNT Reinforced Composite Materials

**DOI:** 10.3390/ma13173718

**Published:** 2020-08-22

**Authors:** Xu-hao Huang, Jian Yang, Iftikhar Azim, Xing-er Wang, Xin Ren

**Affiliations:** 1 State Key Laboratory of Ocean Engineering, Shanghai Jiao Tong University, Shanghai 200240, China; xuhao_huang@sjtu.edu.cn (X.-h.H.); Iftikhar.azim@sjtu.edu.cn (I.A.); matseyo@sjtu.edu.cn (X.-e.W.); 2 Shanghai Key Laboratory for Digital Maintenance of Buildings and Infrastructure, School of Naval Architecture, Ocean and Civil Engineering, Shanghai Jiao Tong University, Shanghai 200240, China; 3 School of Civil Engineering, University of Birmingham, Birmingham B15 2TT, UK; 4 College of Civil Engineering, Nanjing Tech University (Nanjing Tech), Nanjing 211816, China; xin.ren@njtech.edu.cn

**Keywords:** hybrid laminated beam, negative Poisson’s ratio, non-linear vibration, carbon nanotube-reinforced composite, non-linear bending, auxetic materials, Temperature-dependent properties

## Abstract

In the current work, a novel hybrid laminate with negative Poisson’s ratio (NPR) is developed by considering auxetic laminate which is composed of carbon nanotube-reinforced composite (CNTRC) and fiber-reinforced composite (FRC) materials. The maximum magnitude of out-of-plane NPR is identified in the case of (20 ^F^/20 ^C^/−20 ^C^/20 ^C^) _S_ laminate as well. Meanwhile, a method for the geometric non-linear analysis of hybrid laminated beam with NPR including the non-linear bending, free, and forced vibrations is proposed. The beam deformation is modeled by combining higher-order shear-deformation theory (HSDT) and large deflection theory. Based on a two-step perturbation approach, the asymptotic solutions of the governing equations are obtained to capture the linear and non-linear frequencies and load-deflection curves. Moreover, a two-step perturbation methodology in conjunction with fourth-order Runge–Kutta method is employed to solve the forced-vibration problem. Several key factors, such as CNT distribution, variations in the elastic foundation, and thermal stress, are considered in the exhaustive analysis. Theoretical results for some particular cases are given to examine the geometric non-linearity behavior of hybrid beam with NPR as well as positive Poisson’s ratio (PPR).

## 1. Introduction

Materials and structures with negative Poisson’s ratio (NPR) behave in a counter-intuitive manner: when compressed (stretched) in the axial direction, they contract (expand) transversely. The materials and structures that exhibit this feature are also termed as “auxetics” [1]. Lakes [2] first reported NPR behavior in polyurethane (PU) foam with re-entrant structures. Wojciechowski [3] presented the first thermodynamically stable molecular model to study the mechanisms for generating auxetic behavior of solid. NPR was analyzed in systems containing rigid rotating hexamers [4]. Thereafter, analysis on cellular auxetics [5,6], multi-material auxetics [7,8], and auxetic composites [9,10] was carried out. A more systematic review of the development and application for the investigation of auxetic material and structures was reported by Ren et al. [11] and Lakes [12].

In recent years, laminated beams or plates with NPR are developed with more applications as primary structural elements in many fields. Fascinating properties of composite materials with NPR have led researchers to find outstanding applications in the field of aerospace [13,14], automobile industry [15,16], and civil engineering [17,18]. Recently, studies on the design of auxetic structures have propelled to achieve improvements in the mechanical properties such as impact resistance [19,20] and energy absorption [21,22]. On the other hand, with the growing applications of auxetic materials in different industries, developing multi-scale models for design and capturing the non-linear static and dynamic responses of the auxetic laminates under various loading scenarios is critical, when related to the structural design. A series of work has been dedicated to the study of composite beams, in particular, investigations have been carried out on their static and dynamic behavior [23,24,25,26]. However, the value of NPRs (ranging from −0.2 to −1) is not for any particular composite material in real engineering, but is a hypothetical value in a model analysis.

Fiber-reinforced composite (FRC) materials have attracted the attention of researchers, primarily due to the strong anisotropy they offer when designing laminate with NPR. Zhang et al. [27] showed that both the particular stacking sequence and the individual ply material (strongly anisotropic) are essential for a laminate to exhibit NPR. The authors also presented an optimal angle for ply and particular stacking sequences were presented. Evans et al. [28] specially designed a software to predict the effective engineering constants. It was reported that the NPR property can be obtained by designing stacking sequences in the laminated plates. Lempriere [29] measured that the effective Poisson’s ratio (EPR) in orthotropic materials is −0.4, occurring at *θ* = 45° orientation. Clarke et al. [30] reported that the EPR of the laminates show negative values for lay angle in a range between 15° and 30° for a (±θ). It should be noted that when layers are oriented at angles equal in magnitude but with opposite signs, the appropriate + or – sign is used. Herakovich [31] investigated the auxetic characteristics of laminated structure made of graphite-epoxy to determine the value of $v_{13}^{e}$. Such laminates exhibited a very wide range of NPR ranging from a peak of 0.49 for a laminate with ply angle of 90° to as low as −0.21 for laminate with ply angle of (±25)_S_. Hine et al. [32] reported that the out-of-plane Poisson’s ratio reached −1/2 when a high modulus of elasticity carbon fiber is used in the laminates. Matsuda et al. [33] observed that peak values of NPR in carbon fiber-reinforced plastic laminates were around −0.7 when the axis is oriented at 25 ^o^. The influence of Young’s modulus ratio (*E_1_*/*E_2_*), the type of resin and the volume of fraction on the ERP ($v_{13}^{e}$) of an angle-ply [±*θ*]*_2s_* plate was investigated by Harkati et al. [34,35]. It was shown that the NPR of Kevlar and carbon reinforced composite plate is −0.746 at *θ* = 20°. Therefore, it can be concluded form the above discussion that the maximum value of NPR of the laminates greatly depends on the ply orientations and stacking sequences in laminates [36,37,38]. One of the practical applications of such composite structures with NPR is the low-velocity impact resistance [39,40] and static indentation resistance [41]. Alderson and Coenen [39] found that the auxetic FRC laminates showed increased load and energy absorption to failure at low levels of impact energy. Zhou et al. [40] carried out experimental studies on the low-velocity impact response of 3D auxetic composites. They found that 3D auxetic textile composite exhibited excellent impact protective performance compared to 3D non-auxetic textile composite. Coenen and Alderson [41] manufactured auxetic laminates and evaluated their static indentation resistance in comparison with two laminates having near zero and larger positive Poisson’s ratio. The results showed enhancement in load sustained and energy absorption by auxetic laminates.

Carbon nanotubes (CNTs) are extensively used in different fields of industry and research with the development in fabrication technology [42,43,44]. A composite laminate structure made from CNTRCs is prone to exhibit an auxetic feature due to inherent special properties such as strong anisotropy. Furthermore, functionally graded (FG) materials are frequently employed in engineering applications and the laminae with various CNT volume fractions are used to achieve excellent mechanical properties [45]. Based on the studies mentioned above, the auxetic concept of FRC laminates is introduced for designing FRC/CNTRC hybrid laminate with NPR. This hybrid laminate is modeled by placing FRCs in the outermost layers and FG-CNTRC core. Shen was the first investigator to analyze the particular characteristics and behavior of the FG-CNTRC structures at different scales [45]. Following this landmark research, many studies on the forced vibration of FG-CNTRC beams [46,47] and plates [48] were carried out. The free vibration of FG-CNTRC plate were examined by Huu Quoc et al. [49] with a new refined plate theory. Based on Timoshenko beam theory (TBT), the non-linear vibration, forced vibration, and bending behavior of FG-CNTRC beams were performed by Mohammadimehr et al. [50] and Ansari et al. [51]. Considering the matrix cracks, Fan and Wang [52,53] presented an investigation on the non-linear static and dynamic responses of hybrid laminated structures made either from FRCs or CNTRCs. Based on an element-free numerical approach in conjunction with self-consistent model, the effect of matrix cracks on bending and vibration characteristics of hybrid laminated plates were reported by Lei et al. [54,55]. In addition to the applications of FRC/CNTRC hybrid beam or plate, the adoptions of the FRC/CNTRC hybrid concept were later extended to the design of composite blades by Zhang et al. [56,57]. They carried out several investigations exploring the non-linear vibration characteristics of hybrid composited blades. In addition, the design and analysis of the hybrid laminates comprising multi-materials with different strengths have been carried out by [58,59,60,61].

From the literature survey, it can be noted that no research has been carried out to analyze of hybrid beams using FRC/CNTRC materials with NPR at different external conditions. Therefore, the primary objective of the current work is to consider both hybrid configuration and NPR and analyze the static and dynamic characteristics of these auxetic structures. Furthermore, a non-linear model is developed based on the higher-order shear-deformation theory (HSDT) and the Von Kármán large deflection assumption. The motion equations due to thermal stress and reaction force due to foundation are derived. The effects of the CNT material distribution, environment condition, foundation type on the free and forced-vibration feature, and non-linear bending behavior of hybrid laminated beams are investigated in detail. 

## 2. Design of Hybrid Laminate with NPR

As we know that both the ply orientations and stacking sequence have a significant effect on the mechanical response of laminated structures. Therefore, the effective engineering constants are usually used for the convenience of engineers in describing the mechanical behavior of the laminates. Sun et al. [36] and Chen et al. [62] presented a model for EPR for general thick laminates. However, only the extensional response was taken into consideration while the bending and bending-extension coupling characteristics of the laminates were neglected. Therefore, their model fails to provide accurate solutions for EPR of an asymmetric angle-ply laminated plate. Considering the effects of bending and bending-extension coupling, the general solutions of the EPRs for an arbitrary angle-ply laminate are derived in the previous works [63,64,65]. 

For a laminate where the material parameters of the layers are not distributed symmetrically along the section, the general formula of $v_{13}^{e}$ can be expressed as follows:

(1) $$\nu_{13}^{e}=-\left|\begin{array}{cc} A_{13} & B_{11}\\ B_{5\text{-}3} & D \end{array}\right|/\left|\begin{array}{cc} A_{11} & B_{11}\\ B_{5\text{-}1} & D \end{array}\right|,$$

where the matrixes (*A_11_*, *A_13_*, *B_11_*, *B_5-1_*, *B_5-3_*, *D*) are defined in [63,64,65]. 

**Figure 1:**
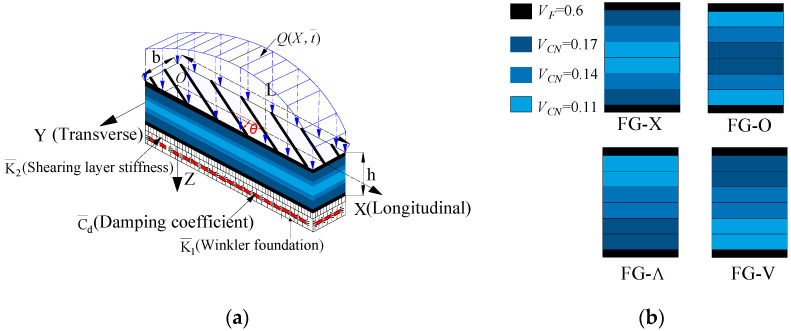
Various types of hybrid laminated beams and reference system: (**a**) Geometry and coordinate system of a hybrid laminated beam on a visco-elastic foundation; (**b**) Four types of CNT distribution in the cross section of a hybrid laminated beam.

For symmetric laminates, the bending-extension coupling stiffnesses *B_ij_* (*i*,*j* = 1,2…6) are zero. The preceding expression simplifies as follows:

(2) $$v_{13}^{e}=\frac{A_{16}(A_{22}A_{36}-A_{23}A_{26})+A_{13}(A_{26}^{2}-A_{22}A_{66})+A_{12}(A_{23}A_{66}-A_{26}A_{36})}{A_{26}^{2}A_{33}-2A_{23}A_{26}A_{36}+A_{23}^{2}A_{66}+A_{22}(A_{36}^{2}-A_{33}A_{66})},$$

where *A_ij_* represents the beam stiffnesses, which are defined in terms of the transformed elastic coefficients (${\bar{C}}_{ij}$)*_k_* as:

(3) $$A_{ij}=\sum_{k=1}^{N}\int_{h_{k-1}}^{h_{k}}{\left({\bar{C}}_{ij}\right)}_{k}dZ,(i,j=1\sim 6),$$

(4) $${\left[{\bar{C}}_{ij}\right]}^{-1}=[{\bar{S}}_{ij}],$$

where the compliance constants (${\bar{S}}_{ij}$) of a laminate whose fibers direction makes an angle *θ* with the direction of X-axis (see Figure 1) and *c* = cos*θ*, s = sin*θ*.

(5) $$\left[\begin{array}{l} {\bar{S}}_{11}\\ {\bar{S}}_{12}\\ {\bar{S}}_{22}\\ {\bar{S}}_{16}\\ {\bar{S}}_{26}\\ {\bar{S}}_{66} \end{array}\right]=\left[\begin{array}{cccc} c^{4} & 2c^{2}s^{2} & s^{4} & c^{2}s^{2}\\ c^{2}s^{2} & c^{4}+s^{4} & c^{2}s^{2} & -c^{2}s^{2}\\ s^{4} & 2c^{2}s^{2} & c^{4} & c^{2}s^{2}\\ 2c^{3}s & 2(cs^{3}-c^{3}s) & -2cs^{3} & -cs(c^{2}-s^{2})\\ 2cs^{3} & 2(c^{3}s-cs^{3}) & -2c^{3}s & cs(c^{2}-s^{2})\\ 4c^{2}s^{2} & -8c^{2}s^{2} & 4c^{2}s^{2} & {\left(c^{2}-s^{2}\right)}^{2} \end{array}\right]\;\left[\begin{array}{c} S_{11}\\ S_{12}\\ S_{22}\\ S_{66} \end{array}\right],$$

(6) $$\left[\begin{array}{c} {\bar{S}}_{13}\\ {\bar{S}}_{23}\\ {\bar{S}}_{33} \end{array}\right]=\left[\begin{array}{ccc} c^{2} & s^{2} & 0\\ s^{2} & c^{2} & 0\\ 0 & 0 & 1 \end{array}\right]\left[\begin{array}{c} S_{13}\\ S_{23}\\ S_{33} \end{array}\right],$$

where the compliance constants of laminate *S_ij_* are given as follow:

(7) $$\left[\begin{array}{ccc} S_{11} & S_{12} & S_{13}\\ S_{22} & S_{23} & S_{33}\\ S_{44} & S_{55} & S_{66} \end{array}\right]=\left[\begin{array}{ccc} 1/E_{11} & -\nu_{12}/E_{11} & -\nu_{13}/E_{11}\\ 1/E_{22} & -\nu_{23}/E_{22} & 1/E_{33}\\ 1/G_{23} & 1/G_{13} & 1/G_{12} \end{array}\right]$$

where the basic material parameters of each layer of laminate are introduced as follow by referring to Figure 1.

*E_ii_*, (*I* = 1,2,3) = Young’s moduli in *i*, (*i*=1,2,3) directions 

*G_ij,_* (*ij* = 12, 13, 23) = Shear moduli in *i-j* planes, respectively

*v*_ij_, *ij* = 12, 13, 23) = Poisson’s ratios (the subscripts *i* and *j* represent the loading and strain directions, respectively).

The theoretical solution presented above can predict the EPR of an arbitrary angle-ply laminates. A systematic investigation of the symmetric hybrid laminates has been carried out. The hybrid laminated beam is designed by placing FRC in the outermost layers and CNTRC in the rest of the layers which gives excellent performance. The lay-ups of the hybrid laminated beam are considered eight-layered and angle-ply (*θ^F^*/*θ^C^*/-*θ^C^*/*θ^C^*)_S_. The subscript/index “S” indicates that the laminate is symmetric and *θ* is the angle of CNT or fiber orientation. Unless otherwise stated, the superscript *F* represents the layer of FRC while the superscript *C* represents the other layers with CNTRC. These configurations are for an identical material and have constant thickness of 0.125 mm and 0.25 mm for FRC and CNT layers, respectively. Several types of CNT distributions are taken into consideration and the type of CNT volume fraction (*V_CN_*) is given. The temperature-related material properties of CNTRCs are predicted by the extended micromechanical model [45] as summarized in Table 1. It should be noted that the thermal expansion coefficients (*α_11_*, *α_22_*) are calculated by Equation (15), which is obtained by the Schapery model [66].

To assess how the distribution and volume fractions of CNT influence the static and dynamic behavior of the beam, we considered five configurations summarized in Table 2. The volume fraction of fiber is fixed as *V*_f_ = 0.6. It differ in the following characteristics: FG-Λ: 0.6/(0.11)_2_/(0.14)_2_/(0.17)_2_/0.6, FG-V: 0.6/(0.17)_2_/(0.14)_2_/(0.11)_2_/0.6, FG-X:(0.6/0.11/0.14/0.17)_S_, FG-O: (0.6/0.17/0.14/0.11)_S_, and uniform distribution (UD): (0.6/0.14/0.14/0.14)_S._ It can be assumed that *G_13_* = *G_23_* = *G_12_*.

The EPR ($v_{13}^{e}$) of the hybrid FRC/CNTRC laminated beam is obtained by adjusting specific ply orientations and stacking sequence. The results in terms of NPR and PPR of hybrid laminated beam and the corresponding ply orientations are presented in Table 3.

## 3. Theoretical Modeling of Hybrid Beams

Laminates consist of layers of composites reinforced with CNTRC and FRC. Consider a hybrid laminated beam composed of eight layers with lamination scheme (*θ*^F^/*θ*^C^/-*θ*^C^/*θ*^C^)_S_ resting on a continuous visco-Pasternak foundation as shown in Figure 1. Figure 1a defines the coordinate system used in development of the hybrid laminated beam analysis. The *XYZ* coordinate system is assumed to have its origin on the middle face of the beam so that the middle surface lies in the *XY*-plane. *Q*(*X*,*t*) is the out-of-plane static or dynamic load. The displacement at a point on the *X*, *Y*, and *Z* directions are $\bar{U}$, $\bar{V}$, and $\bar{W}$, respectively.

The simply supported beam is resting on a three-parameter foundation including the Winkler foundation (${\bar{K}}_{1}$), shearing layer stiffness (${\bar{K}}_{2}$), and damping parameter ${\bar{C}}_{d}$. The reaction force from the visco-Pasternak foundation *P_0_* (*X*,$\bar{t}$) is given by:

(8) $$p_{0}\left(X,\bar{t}\right)={\bar{K}}_{1}\bar{W}\left(X,\bar{t}\right)-{\bar{K}}_{2}\frac{\partial^{2}\bar{W}\left(X,\bar{t}\right)}{\partial X^{2}}+{\bar{C}}_{d}\frac{\partial^{2}\bar{W}\left(X,\bar{t}\right)}{\partial {\bar{t}}^{2}}$$

The method of analysis is based on the HSDT [67] for the laminated beam undergoing large deflection. The effect of the elevated temperature is considered by introducing thermal stress resultants ${\bar{N}}^{T}$,$\;{\bar{M}}^{T}$, and ${\bar{P}}^{T}$ as shown in Appendix A. The motion equations are given as follow:

$$S_{11}\frac{\partial^{4}\bar{W}}{\partial X^{4}}+S_{12}\frac{\partial^{3}{\bar{\Psi }}_{x}}{\partial X^{3}}+\frac{{\bar{B}}_{11}}{{\bar{A}}_{11}}\frac{\partial^{2}{\bar{N}}^{T}}{\partial X^{2}}+\frac{\partial^{2}{\bar{M}}^{T}}{\partial X^{2}}+{\bar{N}}_{x}\frac{\partial^{2}\bar{W}}{\partial X^{2}}+Q\left(X,\bar{t}\right)$$

(9) $$=p_{0}(X,\bar{t})+I_{1}\frac{\partial^{2}\bar{W}}{\partial {\bar{t}}^{2}}+{\hat{I}}_{5}\frac{\partial^{3}{\bar{\Psi }}_{x}}{\partial X\partial {\bar{t}}^{2}}-\frac{4}{3h^{2}}{\hat{I}}_{7}\frac{\partial^{4}\bar{W}}{\partial X^{2}\partial {\bar{t}}^{2}}$$

(10) $$S_{21}\frac{\partial^{3}\bar{W}}{\partial X^{3}}+S_{22}\frac{\partial^{2}{\bar{\Psi }}_{x}}{\partial X^{2}}-S_{23}\left(\frac{\partial \bar{W}}{\partial X}+{\bar{\Psi }}_{x}\right)-S_{26}\frac{\partial {\bar{N}}^{T}}{\partial X}+\frac{\partial {\bar{S}}^{T}}{\partial X}={\tilde{I}}_{3}\frac{\partial^{2}{\bar{\Psi }}_{x}}{\partial {\bar{t}}^{2}}-\frac{4{\tilde{I}}_{5}}{3h^{2}}\frac{\partial^{3}\bar{W}}{\partial X\partial {\bar{t}}^{2}}$$

(11) $${\bar{N}}_{x}=L^{-1}\int_{0}^{L}\left\{\left[\frac{{\bar{A}}_{11}}{2}{\left(\frac{\partial \bar{W}}{\partial X}\right)}^{2}-\frac{4{\bar{E}}_{11}}{3h^{2}}\left(\frac{\partial^{2}\bar{W}}{\partial X^{2}}+\frac{\partial {\bar{\Psi }}_{x}}{\partial X}\right)+{\bar{B}}_{11}\frac{\partial {\bar{\Psi }}_{x}}{\partial X}\right]-{\bar{N}}^{T}\right\}dX$$

where ${\bar{\Psi }}_{x}$ denotes rotation about the longitudinal axes. The reduced stiffnesses of the beam (${\bar{A}}_{11}$,${\bar{B}}_{11}$,$\;{\bar{E}}_{11}$) and the coefficients *S_ij_* and inertias *I_i_* are defined and given in Appendix A. 

The formulae for the forces owing to thermal stress are given as:

(12) $$\left({\bar{N}}^{T},{\bar{M}}^{T},{\bar{P}}^{T}\right)=\sum_{k=1}^{N}b\int_{t_{k-1}}^{t_{k}}{\left[A_{x}\right]}_{k}(1,Z,Z^{3})\Delta T\;dZ$$

(13) $${\bar{S}}^{T}={\bar{M}}^{T}-\frac{4{\bar{P}}^{T}}{3h^{2}}$$

where Δ*T* is the temperature increment from a reference state (*T*_0_ = 300 K), Δ*T* = *T* − *T*_0_. *T* is set as 400 K or 500 K. 

The coefficient *A_x_* is known in terms of the thermal expansion coefficients (*α_11_*, *α_22_*):

(14) $$A_{x}={\bar{Q}}_{11}(c^{2}\alpha_{11}+s^{2}\alpha_{22})+{\bar{Q}}_{12}(s^{2}\alpha_{11}+c^{2}\alpha_{22})+{\bar{Q}}_{16}2cs(\alpha_{11}-\alpha_{22})$$

(15) $$\alpha_{ij}=\{\begin{array}{c} \left(V_{CN}E_{11}^{CN}\alpha_{22}^{CN}+V_{m}E^{m}\alpha^{m}\right)/\left(V_{CN}E_{11}^{CN}+V_{m}E^{m}\right)\;ij=11\;\\ (1+\nu_{12}^{CN})V_{CN}\alpha_{22}^{CN}+(1+\nu^{m})V_{m}\alpha^{m}-\nu_{12}\alpha_{11}\;ij=22 \end{array}$$

where *α_ij_* (*ij* = *11*,*22*) are the thermal expansion coefficients and subscripts 11 and 22 denote the longitudinal and transverse directions, respectively. The formulae for calculating the *α_11_* and *α_22_* are obtained from [66]. 

It should be noted that Equation (11) represent restricted boundary conditions. It is suitable for a beam with immovable boundary condition i.e., the longitudinal displacement is equal to zero at both ends of the beam. It should be noted that this immovable boundary condition is unacceptable for the compressive post-buckling analysis of beam.

The non-linear motion equations for the vibration can be solved by a two-step perturbation approach proposed by Shen [68,69]. By deriving the dimensionless forms of the motion Equations (9)–(11), we get:

(16) $$\begin{array}{c} \gamma_{11}\frac{\partial^{4}W}{\partial x^{4}}-\gamma_{12}\frac{\partial^{3}\Psi_{x}}{\partial x^{3}}-\pi \left\{\int_{0}^{\pi }\left[\frac{\gamma_{13}}{2}{\left(\frac{\partial W}{\partial x}\right)}^{2}+\gamma_{14}\frac{\partial \Psi_{x}}{\partial x}-\gamma_{15}\frac{\partial^{2}W}{\partial x^{2}}\right]dx\right\}\frac{\partial^{2}W}{\partial x^{2}}+C_{1}\frac{\partial^{2}W}{\partial x^{2}}-\gamma_{16}\frac{\partial^{2}N^{T}}{\partial x^{2}}\\ -C_{2}\frac{\partial^{2}M^{T}}{\partial x^{2}}=\lambda_{q}-(K_{1}W-K_{2}\frac{\partial^{2}W}{\partial x^{2}}+C_{d}\frac{\partial W}{\partial t})+\gamma_{17}\frac{\partial^{2}W}{\partial t^{2}}+\gamma_{18}\frac{\partial^{3}\Psi_{x}}{\partial x\partial t^{2}}+\gamma_{19}\frac{\partial^{4}W}{\partial x^{2}\partial t^{2}} \end{array}$$

(17) $$\gamma_{21}\frac{\partial^{3}W}{\partial x^{3}}-\gamma_{22}\frac{\partial^{2}\Psi_{x}}{\partial x^{2}}+\gamma_{23}\left(\frac{\partial W}{\partial x}+\Psi_{x}\right)-\gamma_{26}\frac{\partial N^{T}}{\partial x}-C_{3}\frac{\partial S^{T}}{\partial x}=\gamma_{28}\frac{\partial^{2}\Psi_{x}}{\partial t^{2}}+\gamma_{29}\frac{\partial^{3}W}{\partial x\partial t^{2}}$$

where

(18) $$\left(C_{1},C_{2},C_{3}\right)=\left(\gamma_{T1},\gamma_{T3},\gamma_{T3}-\gamma_{T6}\right)\Delta T$$

The non-dimensional parameters mentioned above can be written as follow:

(19) $$\begin{array}{c} \left(x,W,\Psi_{x}\right)=\left(\frac{\pi X}{L},\frac{\bar{W}}{L},\frac{{\bar{\Psi }}_{x}}{\pi }\right),\;(N_{x},M_{x},\;P_{x})=\frac{L^{2}}{\pi^{2}h{\bar{D}}_{11}}\left(h{\bar{N}}_{x},{\bar{M}}_{x},\;\frac{4}{3h^{2}}{\bar{P}}_{x}\right),\\ (K_{1},K_{2})=\frac{L^{2}}{\pi^{2}{\bar{D}}_{11}}\left(\frac{L^{2}}{\pi^{2}}{\bar{K}}_{1},\;{\bar{K}}_{2}\right),\;(k_{1},k_{2})=\frac{L^{2}}{E_{0}I}\left(L^{2}{\bar{K}}_{1},\;{\bar{K}}_{2}\right),\\ (\gamma_{11},\;\gamma_{12},\;\gamma_{21},\;\gamma_{22})=\frac{1}{{\bar{D}}_{11}}\left(-S_{11},\;S_{12},\;-S_{21},\;S_{22}\right),\;\gamma_{23}=\frac{L^{2}}{\pi^{2}{\bar{D}}_{11}}S_{23},\\ \left(\gamma_{13},\;\gamma_{14},\;\gamma_{15})=\frac{L}{\pi^{2}{\bar{D}}_{11}}(L{\bar{A}}_{11},{\bar{B}}_{11}-\frac{4}{3h^{2}}{\bar{E}}_{11},\;\frac{4}{3h^{2}}{\bar{E}}_{11}\right),\;(\gamma_{16},\;\gamma_{26})=\frac{1}{{\bar{A}}_{11}L}\left({\bar{B}}_{11},\;{\bar{B}}_{11}-\frac{4}{3h^{2}}{\bar{E}}_{11}\right),\\ (\gamma_{17},\gamma_{18},\gamma_{19},\gamma_{28},\;\gamma_{29})=-\;\left(\frac{I_{1}L^{2}}{\pi^{2}}{\hat{I}}_{5},\;-\frac{4}{3h^{2}}{\hat{I}}_{7},\;{\tilde{I}}_{3},\;-\frac{4}{3h^{2}}{\tilde{I}}_{5}\right)\frac{bE_{0}}{\rho_{0}{\bar{D}}_{11}},\;t=\frac{\pi \;\bar{t}}{L}\sqrt{\frac{E_{0}}{\rho_{0}}},\;\gamma_{T1}=\frac{L^{2}A_{x}^{T}}{\pi^{2}{\bar{D}}_{11}},\\ \omega_{L}=\Omega_{L}\frac{L}{\pi }\sqrt{\frac{\rho_{0}}{E_{0}}},\;\left(\gamma_{T3},\;\gamma_{T6})=\frac{L^{2}}{\pi^{2}h{\bar{D}}_{11}}(D_{x}^{T},\;\frac{4}{3h^{2}}F_{x}^{T}\right),\;\lambda_{q}=\frac{QL^{3}}{\pi^{4}{\bar{D}}_{11}},\;C_{d}={\bar{C}}_{d}\frac{a^{3}}{\pi^{3}D_{11}^{*}}\sqrt{\frac{E_{0}}{\rho_{0}}} \end{array}$$

in which *E*_0_ = *E*^m^ and *ρ^0^* = *ρ^m^* are the elastic modulus and density of the matrix. $A_{x}^{T}$, $D_{x}^{T}$, $F_{x}^{T}$ are the functions of thickness and *A*_x_ can be defined by:

(20) $$(A_{x}^{T},\;D_{x}^{T},\;F_{x}^{T})=b\sum_{k=1}^{N}\int_{t_{k-1}}^{t_{k}}{\left[A_{x}\right]}_{k}(1,Z,Z^{3})dZ$$

## 4. Free Vibration Analysis

For non-linear vibration problem, the solutions for Equations (16) and (17) consist of an additional displacement term and initial displacement term as a result of the varying temperature. The initial deflection under the thermal loading for these structures are reported from Shen [63]. By considering, *τ* = *εt*, the solution equations can be expanded as a function with a small perturbation parameter *ε^j^* (*j* = 1,2,3,….) as given below:

(21) $$\begin{array}{c} \Psi_{x}(x,\tau ,\varepsilon )=\varepsilon \psi_{x1}(x,\tau )+\varepsilon^{2}\psi_{x2}(x,\tau )+\varepsilon^{3}\psi_{x3}(x,\tau )+\cdot \cdot \cdot \\ \Psi_{x}(x,\tau ,\varepsilon )=\varepsilon \psi_{x1}(x,\tau )+\varepsilon^{2}\psi_{x2}(x,\tau )+\varepsilon^{3}\psi_{x3}(x,\tau )+\cdot \cdot \cdot \\ \lambda_{q}(x,\tau ,\varepsilon )=\varepsilon \lambda_{q1}(x,\tau )+\varepsilon^{2}\lambda_{q2}(x,\tau )+\varepsilon^{3}\lambda_{q3}(x,\tau )+\cdot \cdot \cdot \end{array}$$

The boundary conditions for *τ* = 0 can be expressed as:

(22) $$W{|}_{\tau =0}=\Psi_{x}{|}_{\tau =0}=0,\;\frac{\partial W}{\partial \tau }{|}_{\tau =0}=\frac{\partial \Psi_{x}}{\partial \tau }{|}_{\tau =0}=0,(\mathrm{simply}\;\mathrm{supported}).$$

If we substitute *τ* = *εt* and Equation (21) into Equations (16) and (17) and collect all the terms of the same order of *ε^i^* (*i* = 1,2,3,…), we will get a set of perturbation equations. The solution of Euler-Bernoulli beam with simply supported conditions can be used for perturbation equations with *ε^i^* (*i* = 1)

(23) $$w_{1}(x,\tau )=A_{10}^{\left(1\right)}(\tau )\sin mx$$

Then, the equations with *ε^j^* (*j* = 1,2,3,...) can be solved step by step, we will get:

(24) $$W(x,t,\varepsilon )=\varepsilon A_{10}^{\left(1\right)}(t)\sin mx+O(\varepsilon^{4})$$

(25) $$\Psi_{x}(x,t,\varepsilon )=\varepsilon B_{10}^{\left(1\right)}(t)\cos mx+\varepsilon^{3}B_{10}^{\left(3\right)}(t)\cos mx+O(\varepsilon^{4})$$

(26) $$\begin{array}{c} \lambda_{q}(x,t,\varepsilon )=\left(\varepsilon {\ddot{A}}_{10}^{\left(1\right)}(t)g_{30}+\varepsilon A_{10}^{\left(1\right)}(t)g_{31}\right)\sin mx+\left({\left(\varepsilon A_{10}^{\left(1\right)}(t)\right)}^{2}g_{321}+(\varepsilon A_{10}^{\left(1\right)})(\varepsilon A_{10}^{*})g_{322}\right)\sin mx\\ +{\left(\varepsilon A_{10}^{\left(1\right)}(t)\right)}^{3}g_{33}\sin mx+O\left(\varepsilon^{4}\right) \end{array}$$

Introducing expression *τ* = *t* and *ε*$A_{10}^{\left(1\right)}$=*W_m_* in Equation (26) and applying Galerkin procedure yielded Equation (26) which can be re-written as:

(27) $$g_{30}\frac{d^{2}(W_{m})}{dt^{2}}+g_{c}\frac{d(W_{m})}{dt}+g_{31}(W_{m})+g_{32}{\left(W_{m}\right)}^{2}+g_{33}{\left(W_{m}\right)}^{3}-{\bar{\lambda }}_{q}\left(t\right)=0$$

The numerical solution of Equation (27) corresponds to the forced-vibration response of the structure. It can be converted to a free vibration problem by setting the uniform load to zero. Therefore, the solution of the differential equation with $g_{c}={\bar{\lambda }}_{q}$(*t*) = 0 can be obtained as follow:

(28) $$\omega_{NL}=\omega_{L}{\left(1+\frac{9g_{31}g_{33}-10g_{32}^{2}}{12g_{31}^{2}}A^{2}\right)}^{1/2}$$

(29) $$\omega_{L}={\left(g_{31}/g_{30}\right)}^{1/2}$$

where *ω_NL_* and *ω*_L_ represent the dimensionless non-linear and linear frequency, respectively. *A* = *W_m_* = ${\bar{W}}_{m}$/*L*. ${\bar{W}}_{m}$ is the amplitude of deflection. According to Equation (19), the corresponding linear frequency can be expressed as Ω** = *ω_L_*(*π*/*L*)(*E*_0_/*ρ*_0_)^1/2^. The details of *g_30_*, *g_31_*, *g_32_*, and *g_33_* are described in Appendix B.

**Figure 2:**
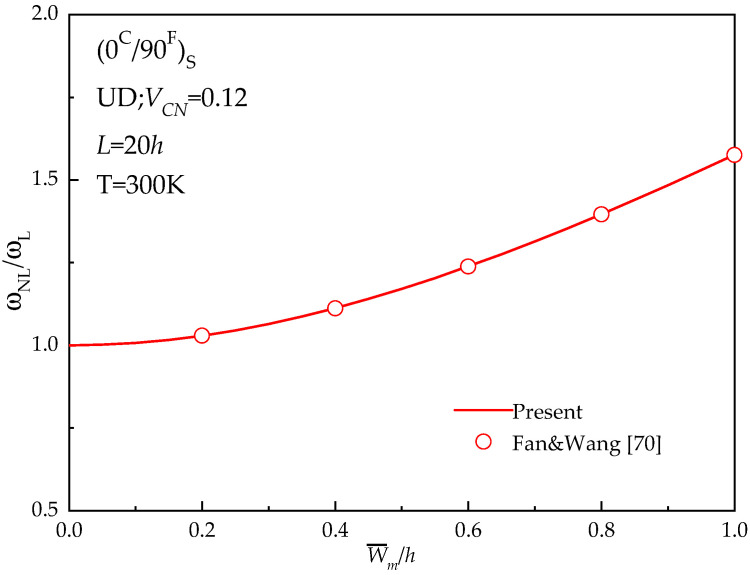
Comparison of *ω_NL_*/*ω_L_* for angle-ply (0 ^C^/90 ^F^) _S_ hybrid laminated beam.

For the purpose of verification, Table 4 and Figure 2 show the comparison of the solutions obtained by the present method and the method proposed by Fan & Wang [70] with the present solutions of a hybrid laminated beam. The reinforced material of the hybrid beam consists of two parts: (1) for FRC, $E_{11}^{F}$ = 233.05 GPa, $v_{12}^{f}$ = 0.2,$\;E_{22}^{F}$ = 23.1 GPa, *ρ^f^* = 1750 kg/m³,$G_{12}^{F}$ = 8.96 GPa, (2) for CNTRC, $E_{11}^{CN}$ = 5646.6 GPa, $v_{12}^{CN}$ = 0.175,$\;E_{22}^{CN}$ = 7080.0 GPa, *ρ^CN^* = 1400 kg/m³ and $\;G_{12}^{CN}$ = 1944.5 GPa. The material properties for the same matrix are *E^m^* = 2.5 GPa, *ρ^m^* = 1150 kg/m^3^, and ν^m^ = 0.34. The value of geometric parameters used are: *L* = 20 h, *h* = 0.5mm. 

Table 4 compares the fundamental frequencies $\tilde{\Omega }\;$ = Ω**(*L*^2^/*h*)$\sqrt{\rho_{0}/E_{0}}$ for (*θ*^C^/90^F^)_S_ hybrid beam with different values of *V*_CN_ and the angle of the CNT orientation with *θ*^C^ = 15°, 30°, and 45°. It can be found that the predicted frequencies tally reasonably well with those results obtained from Fan & Wang [70]. Meanwhile, the non-linear vibration of a (0^C^/90^F^)_S_ hybrid laminated beam is validated with Fan &Wang study, as plotted in Figure 2. It can be observed from Figure 2 that the results in terms of frequency ratio obtained from the presented model are very close to the predictions made by Fan & Wang [70]. Based on the above discussion, it can be concluded that the proposed model is accurate to analyze free vibration of the hybrid laminated beams. 

In the following, a detailed investigation of the vibration of hybrid laminated beams has been carried out. The effect of temperature *T*, distribution pattern, and the foundation constants (*k_1_*, *k_2_*) on the free vibration of hybrid laminated beams are scrutinized. In the following study, the angle-ply (25 ^F^/20 ^C^/-20 ^C^/20 ^C^) _S_ and (25 ^F^/90 ^C^/-90 ^C^/90 ^C^) _S_ hybrid laminated beams are adopted. 

The effect of temperature *T* on the first four frequencies ${\tilde{\Omega }}_{i}$ (*i* = 1, 2, 3, 4) of hybrid laminated beams with five different distribution pattern is investigated in Table 5. It is observed that increase in temperature cause decrease in the frequencies of both the angle-ply hybrid laminated beams. In addition, the temperature effect of the first-order frequency is more significant than on the other order frequencies. Consequently, the influence of degradation caused by thermal stress should be taken into consideration in the design and service of hybrid laminated beams.

Table 6 shows the effects of foundation constants on the hybrid laminated beam with length/thickness ratio of 20. Three different foundation coefficients (*k*_1_, *k*_2_) are (10^3^, 10), (10^3^, 0), and (0, 0), respectively. It should be noted that structures with (*k*_1_, *k*_2_) = (0, 0) are selected for comparison. As can be seen from Table 6, the natural frequencies of the hybrid laminated beam for higher (*k*_1_, *k*_2_) are more than the other cases.

**Figure 3:**
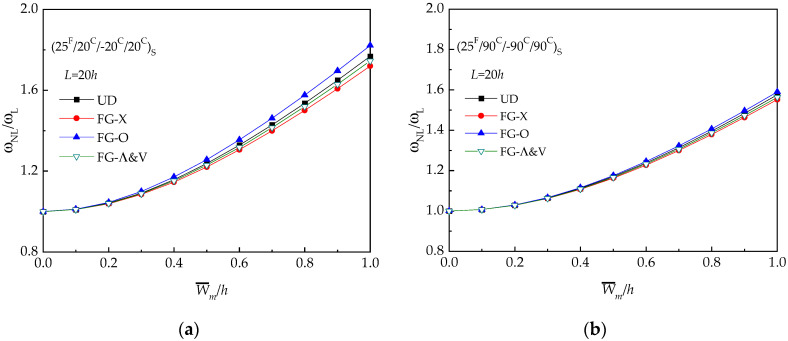
Frequency ratio (*ω_NL_*/*ω_L_*) versus dimensionless deflection ($\bar{W}/h$) of hybrid laminated beams: (**a**) (25 ^F^/20 ^C^/−20 ^C^/20 ^C^) _S_; (**b**) (25 ^F^/90 ^C^/−90 ^C^/90 ^C^) _S._

The frequency ratio–deflection curves for all the five types of hybrid laminated beams are described in Figure 1. As expected, FG-X has the highest frequency ratio while FG-O has the lowest frequency ratio. At the room temperature, FG-Λ and FG-V show similar higher amplitude frequency (see Figure 3). 

**Figure 4:**
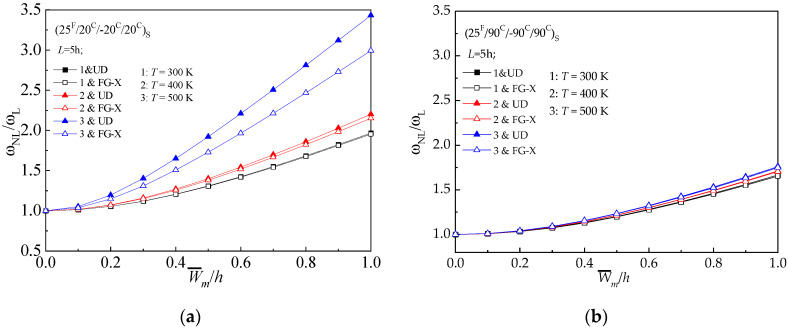
Frequency ratio (*ω_NL_*/*ω_L_*) versus dimensionless deflection ($\bar{W}/h$) of hybrid beams with various temperature conditions (*T* = 300 K, 400 K, 500 K): (**a**) (25 ^F^/20 ^C^/−20 ^C^/20 ^C^)_S_; (**b**) (25 ^F^/90 ^C^/−90 ^C^/90 ^C^) _S._

The influence of temperature field on the non-linear vibration behavior of UD and FG-X hybrid laminated beam with NPR is examined by considering three different temperature values (*T* = 300 K,400 K,500 K). Due to the presence of thermal stress, increase in the temperature leads to a higher frequency ratio, as shown in Figure 4. It is found that (25 ^F^/20 ^C^/−20 ^C^/20 ^C^) _S_ laminated beams are more sensitive to the temperature effect than those with (25 ^F^/90 ^C^−90 ^C^/90 ^C^) _S_.

**Figure 5:**
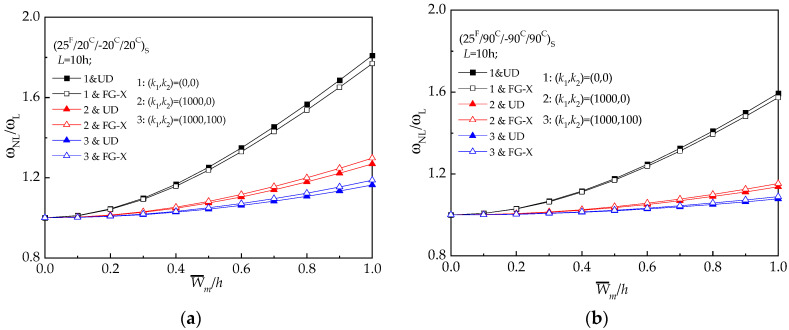
Frequency ratio versus dimensionless deflection of hybrid beams with various elastic foundation constants at reference temperature (*T* = 300 K): (**a**) (25 ^F^/20 ^C^/−20 ^C^/20 ^C^) _S_; (**b**) (25 ^F^/90 ^C^/−90 ^C^/90 ^C^)_S._

The effect of the foundation coefficients on the resulting lager amplitude vibration frequency of UD and FG-X are examined in Figure 5, for a member with *L* = 10 *h*. From Figure 5, it is confirmed that the beam becomes more rigid as the foundation coefficients of the beam increases. Hence, the frequency ratio of the UD and FG-X increases.

## 5. Forced-Vibration Analysis

In this section, the dynamic response of hybrid laminated beams is investigated by considering uniform load *Q* which is dependent on time as plotted in Figure 1. We need to determine the relationship between central deflection and time. Hence, a numerical procedure for solving the second order differential Equation (27) is employed here. Given the initial value *W_m_* (*t_0_*) and ${\dot{\tilde{W}}}_{m}\;$(*t_0_*) at initial time *t_0_* = 0, Equation (27) can be solved to obtain the central deflection-time relationship for the beam under applied load by employing the fourth-order Runge–Kutta method. Here, it should also be noted that the initial deflection for FG-Λ or FG-V will be triggered because of thermal stress.

**Figure 6:**
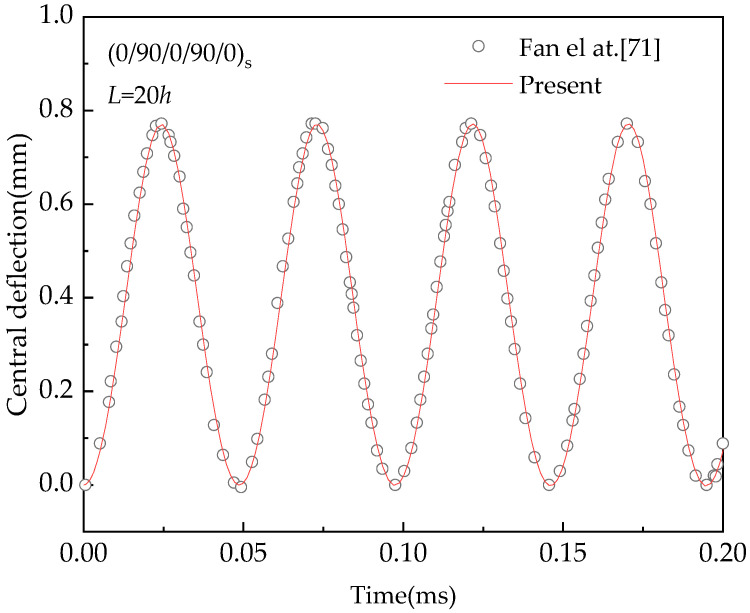
Comparison of forced-vibration curves for angle-ply (0/90/0/90/0)_S_ GRC laminated beam under a sudden uniform load.

In the following, the time response history of the verification analyses is presented. Figure 6 shows the comparison of the solution of a cross-ply (0/90/0/90/0)_S_ graphene-reinforced composite (GRC) laminated beam under a sudden load *Q* = 12 Mpa by the method presented by Fan et al. [71]. and the method presented in this study. The dimensions and material properties of the GRC layer are as follows: *L* = 20 *h =* 60 mm, $E_{11}^{G}$*=* 1.812 TPa, $E_{22}^{G}$= 1.87 TPa, $G_{12}^{G}$= 0.683 TPa, $v_{12}^{G}$= 0.177, *ρ*^G^ = 4118 kg/m³, *E^m^* = 2.5 GPa, *ρ*^m^ = 1150 kg/m³. *v^m^* = 0.34. The subscripts, *G* and *m*, represent the graphene and matrix, respectively. In the forced-vibration stage (time range 0 to 0.2 ms), the central deflection-time curve is described in Figure 6. It is found from Figure 6 that the forced response predicted from the proposed model follows the same trend and agrees well with that provided by Fan et al. [71]. 

**Figure 7:**
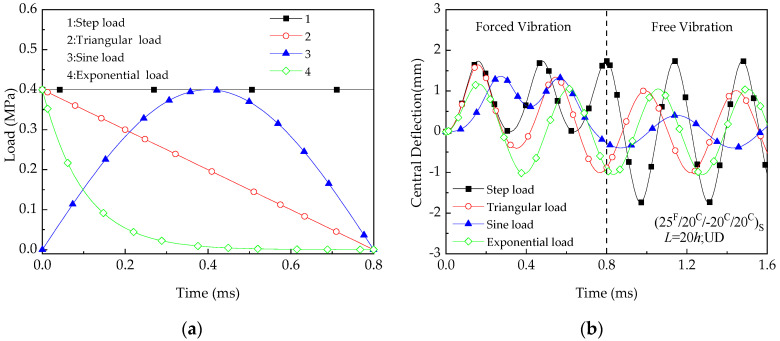
Central deflection versus time of hybrid beams under different dynamic loading scenarios (T = 300 K): (**a**) Type of dynamic loads; (**b**) Vibration curve.

Let us examine the forced-vibration behavior by applying the proposed method to the cases (25 ^F^/20 ^C^/−20 ^C^/20 ^C^) _S_. Figure 7 exhibits the forced-vibration curves of the uniform distribution (UD) beam under the reference temperature (*T* = 300 K). Four different dynamic loads are taken into consideration. The values of the parameters used are *L =* 20 *h*, *h* = 1.75 mm. In the forced-vibration region (time range 0 to 0.8ms), it can be observed that the step load leads to highest deflection among the four while lowest deflection occurs at the exponential load. At time range of 0.8 to 1.6ms, the free vibration behavior of hybrid laminated beams with an initial deflection and velocity is triggered resulting of the dynamic load is released. Please note that a sudden uniform load is taken as the applied load in the parametric study.

**Figure 8:**
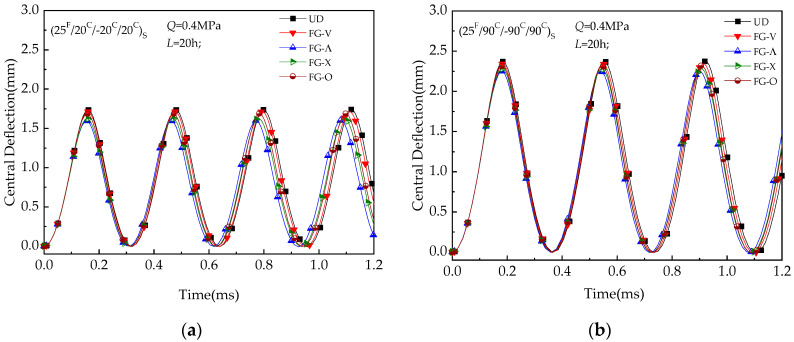
Central deflection versus time (*t*) of hybrid laminated beams: (**a**) (25 ^F^/20 ^C^/−20 ^C^/20 ^C^) _S_; (**b**) (25 ^F^/90 ^C^/−90 ^C^/90 ^C^) _S._

Figure 8 depicts central deflection versus time for UD and FG-CNTRC beam under the reference temperature (*T* = 300 K). Five different distribution patterns designed above are considered. The value of load amplitude is taken as *Q* = 0.4 MPa. Figure 8 shows deflection-time curves among the above designed distributing patterns. The curves of the UD pattern are used at the same temperature for reference. It can be observed that the lowest deflection is obtained by FG-Λ beam as the UD plate has the highest deflection. Thus, for the next parametric analysis, UD and FG-Λ are considered/studied.

**Figure 9:**
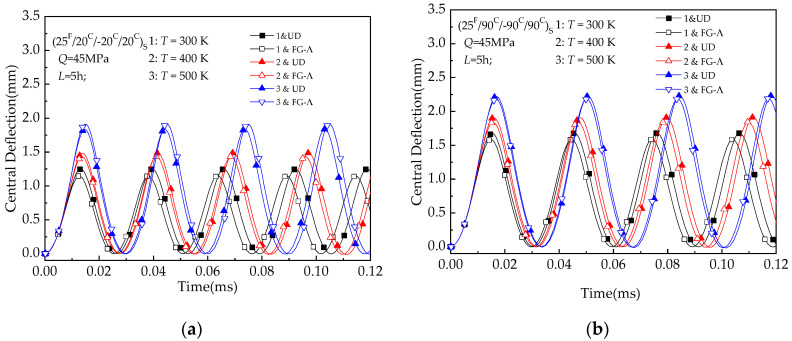
Deflection versus time (*t*) of hybrid laminated beams with various temperature conditions (T = 300 K, 350 K, 400 K): (**a**)(25 ^F^/20 ^C^/−20 ^C^/20 ^C^) _S_; (**b**) (25 ^F^/90 ^C^/−90 ^C^/90 ^C^) _S._

The above approach is also used to investigate the influence of changing the temperature field on UD and FG-Λ beams. Figure 9 shows the influence of the thermal stress on UD and FG-Λ beams. The applied load is 45 MPa for (25 ^F^/20 ^C^/−20 ^C^/20 ^C^) _S_ and (25 ^F^/90 ^C^/−90 ^C^/90 ^C^) _S_ with *L* = 5 h. Incremental temperatures are marked on these curves. The curves show that the amplitude and period of response increase with increase in temperature. This is again attributed to the fact that the development of thermal resultants reduces the overall plate stiffness.

**Figure 10:**
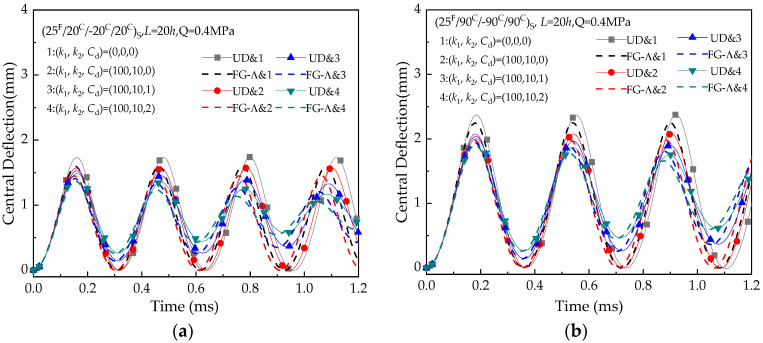
Deflection versus time (*t*) of hybrid laminated beams with various foundation constants at the reference temperature (T = 300 K): (**a**) (25 ^F^/20 ^C^/−20 ^C^/20 ^C^) _S_; (**b**) (25 ^F^/90 ^C^/−90 ^C^/90 ^C^) _S._

Figure 10 shows the maximum deflection with time variation of hybrid beams (25 ^F^/20 ^C^/−20 ^C^/20 ^C^) _S_ and (25 ^F^/90 ^C^/−90 ^C^/90 ^C^)_S_ for different foundations stiffnesses ((*k*_1_, *k*_2_ *C*_d_) = (0, 0, 0), (10^2^, 0, 0), (10^2^, 10, 1), (10^2^, 10,2)). Beams without foundation (*k*_1_ = *k*_2_ = *C*_d_ = 0) are selected as a reference. The results confirm that the foundation stiffness decrease the transverse deflection. Moreover, the deflection of beams on the viscoelastic foundation decreases with increasing time.

## 6. Non-Linear Bending Analysis

In this section, non-linear bending response of hybrid laminated beams with NPR and PPR will be discussed in detail. For this purpose, we will determine relationship between applied pressure and deflection of the beam. For the static analysis, this relationship is independent of the change in time (*t*). Therefore, the transverse load is modified to be uniformly distributed, and *Q* (*X*, *t*) = *Q* (*X*) = *q_0_*. *W* is independent of time. Under these assumptions, Equations (16) and (17) can be simplified as:

$$\gamma_{11}\frac{\partial^{4}W}{\partial x^{4}}-\gamma_{12}\frac{\partial^{3}\Psi_{x}}{\partial x^{3}}-\pi \left\{\int_{0}^{\pi }\left[\frac{\gamma_{13}}{2}{\left(\frac{\partial W}{\partial x}\right)}^{2}+\gamma_{14}\frac{\partial \Psi_{x}}{\partial x}-\gamma_{15}\frac{\partial^{2}W}{\partial x^{2}}\right]dx\right\}\frac{\partial^{2}W}{\partial x^{2}}+C_{1}\frac{\partial^{2}W}{\partial x^{2}}$$

(30) $$-\gamma_{16}\frac{\partial^{2}N^{T}}{\partial x^{2}}-C_{2}\frac{\partial^{2}M^{T}}{\partial x^{2}}=\lambda_{q}-(K_{1}W-K_{2}\frac{\partial^{2}W}{\partial x^{2}})$$

(31) $$\gamma_{21}\frac{\partial^{3}W}{\partial x^{3}}-\gamma_{22}\frac{\partial^{2}\Psi_{x}}{\partial x^{2}}+\gamma_{23}\left(\frac{\partial W}{\partial x}+\Psi_{x}\right)-\gamma_{26}\frac{\partial N^{T}}{\partial x}-C_{3}\frac{\partial S^{T}}{\partial x}=0$$

A perturbation approach is also used to derive the solutions of Equations (30) and (31). The solution equations can be expanded as a function with a small perturbation parameter *ε^j^* (*j* = 1,2,3,…).

(32) $$\begin{array}{c} W(x,\varepsilon )=\sum_{j=1}\varepsilon^{j}w_{j}(x)\\ \Psi_{x}(x,\varepsilon )=\sum_{j=1}\varepsilon^{j}\psi_{xj}(x)\\ \lambda_{q}(x,\varepsilon )=\sum_{j=1}\varepsilon^{j}\lambda_{j}(x) \end{array}$$

Following the perturbation solutions procedure, the solutions for the equations with the first order *ε* can be assumed as:

(33) $$w_{1}(x)=A_{10}^{\left(1\right)}\sin mx$$

The thermal load can be expanded as the Fourier modes as:

(34) $$\left[\begin{array}{c} M_{x}^{T}\\ S_{x}^{T} \end{array}\right]=\sum_{k=1,3,\ldots }\frac{-1}{k}\sin kx\left[\begin{array}{c} M_{x}^{\left(1\right)}\\ S_{x}^{\left(1\right)} \end{array}\right]$$

(35) $$\left(M_{x}^{\left(1\right)},S_{x}^{\left(1\right)}\right)=\frac{4}{\pi }\left(\gamma_{T3},\gamma_{T3}-\gamma_{T6}\right)\Delta T$$

For the free vibration analysis, the half-wavelength (*m*) can be assumed as *m* = 1,2,3…, whereas, for the bending study *m* is adopted as 1. Substituting Equations (32) and (35) in the motion equations, we get the asymptotic solution as:

(36a) $$W(x,\varepsilon )=\varepsilon A_{10}^{\left(1\right)}\sin mx+O\left(\varepsilon^{4}\right)$$

(36b) $${\tilde{\Psi }}_{x}(x,\varepsilon )=\varepsilon B_{10}^{\left(1\right)}\cos mx+O\left(\varepsilon^{4}\right)$$

and

(37) $$\lambda_{q}(x,\varepsilon )=\lambda_{q1}(A_{10}^{\left(1\right)}\varepsilon )+\lambda_{q2}{\left(A_{10}^{\left(1\right)}\varepsilon \right)}^{2}+\lambda_{q3}{\left(A_{10}^{\left(1\right)}\varepsilon \right)}^{3}+O\left(\varepsilon^{4}\right)$$

Equations (36) and (37) are the functions of $A_{10}^{\left(1\right)}$. In Equations (36) and (37), (*ε*$A_{10}^{\left(1\right)}$) is taken as the second perturbation parameter related to the dimensionless maximum deflection *W*_m_. From Equations (36a) and (37), the relationships between the load and central displacement are obtained as:

(38) $$\frac{q_{0}L^{3}}{\pi^{4}{\bar{D}}_{11}}=A_{W}^{\left(0\right)}+A_{W}^{\left(1\right)}\left(\frac{{\bar{W}}_{m}}{L}\right)+A_{W}^{\left(2\right)}{\left(\frac{{\bar{W}}_{m}}{L}\right)}^{2}+A_{W}^{\left(3\right)}{\left(\frac{{\bar{W}}_{m}}{L}\right)}^{3}+\ldots$$

in which

(39) $$\begin{array}{c} A_{W}^{\left(0\right)}=-\;\left[\gamma_{T3}-(\gamma_{T3}-\gamma_{T6})\frac{\gamma_{12}}{\gamma_{22}+\gamma_{23}}\right]\Delta T,\\ A_{w}^{\left(1\right)}=\frac{\pi }{4}\left[\left(\gamma_{11}-\gamma_{12}\frac{\gamma_{21}-\gamma_{23}}{\gamma_{22}+\gamma_{23}}\right)+(K_{1}+K_{2})-\gamma_{T1}\Delta T\right],\\ A_{W}^{\left(2\right)}=\frac{\pi^{2}}{2}\left(\gamma_{15}-\gamma_{14}\frac{\gamma_{21}-\gamma_{23}}{\gamma_{22}+\gamma_{23}}\right),\\ A_{W}^{\left(3\right)}=\frac{\pi^{3}}{16}\gamma_{13}, \end{array}$$

**Figure 11:**
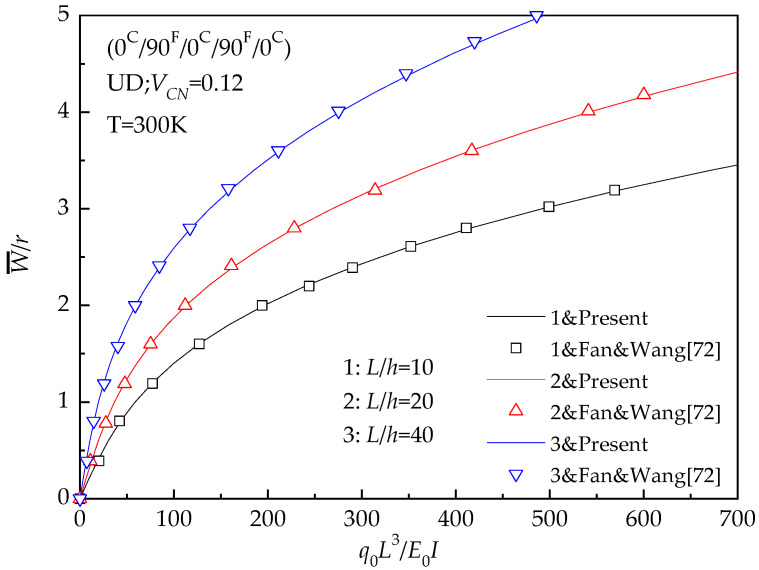
Comparisons of load-deflection relationships of a hybrid laminated beam.

The non-linear bending of a hybrid laminated beam with cross-ply (0 ^C^/90 ^F^/0 ^C^/90 ^F^/0 ^C^) under a uniformly distributed load is studied in the current analysis. Both the ends of the beam are taken as simply supported. The same analysis has been reported by Fan & Wang [72]. Constituent materials of the beam are similar to the cases of hybrid beam listed in Table 4. Present dimensionless deflections are compared with those of Fan & Wang [72] for the hybrid laminated beam with *L*/*h* = 10, 20, 40. For the sake of consistency, we have used *q_o_L^3^/E_0_I*, which is adopted by Fan & Wang [72]. Nevertheless, *q_o_L^4^/E_0_I* is non-dimensional load which is adopted in this paper. According to curves plotted in Figure 11, similar trend and good accordance may be observed between the results.

**Figure 12:**
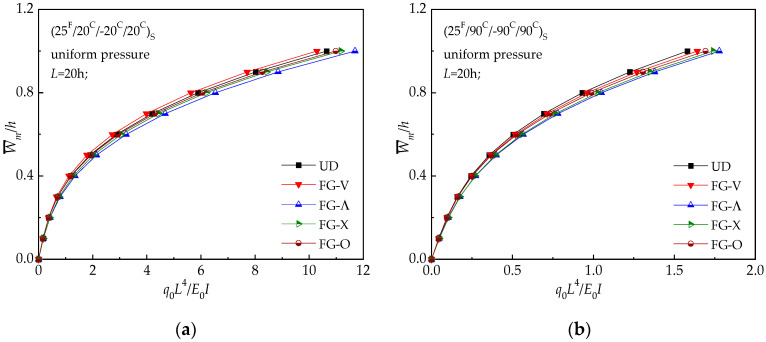
Load-deflection relationships of hybrid laminated beams: (**a**) (25 ^F^/20 ^C^/−20 ^C^/20 ^C^) _S_; (**b**) (25 ^F^/90 ^C^/−90 ^C^/90 ^C^) _S._

Figure 12 illustrates the dimensionless load-deflection curves for UD and FG beam under a uniform pressure. Five different distribution patterns designed above are considered. ${\bar{W}}_{m}$/*h* and *q_o_L^4^/E_0_I* represent the dimensionless defection and load, respectively. Figure 12 shows the deflection-time curves among the above-mentioned distributing patterns. The curve of the UD pattern is used at the same temperature for reference. UD beam has the maximum central deflection as compared with the other distribution patterns while FG-Λ beam has the minimum central displacement. Thus, for the next comprehensive studies, UD and FG-Λ are taken as the case studies.

**Figure 13:**
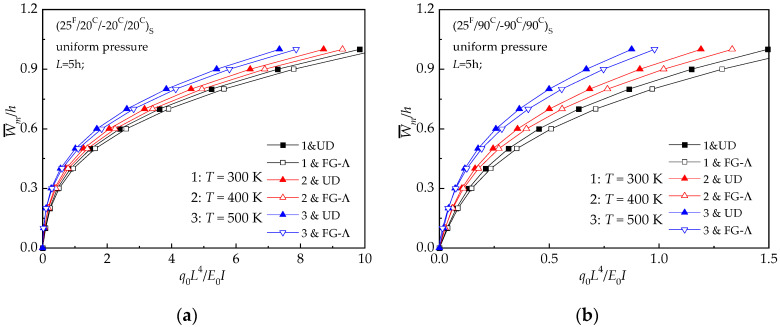
Load-deflections relationships of hybrid laminated beams at various temperatures: (**a**) (25 ^F^/20 ^C^/−20 ^C^/20 ^C^) _S_; (**b**) (25 ^F^/90 ^C^/−90 ^C^/90 ^C^) _S._

Figure 13 demonstrates the influence of the changing temperature field on the bending response of UD and FG-Λ with the above-mentioned conditions and *L* = 5 *h*. It may be concluded from Figure 13 that the central deflection increases with a rise in temperature. It is also observed that (25 ^F^/20 ^C^/−20 ^C^/20 ^C^) _S_ laminated beams are less sensitive to variations in the temperature than (25 ^F^/90 ^C^/−90 ^C^/90 ^C^) _S_ laminated beam.

**Figure 14:**
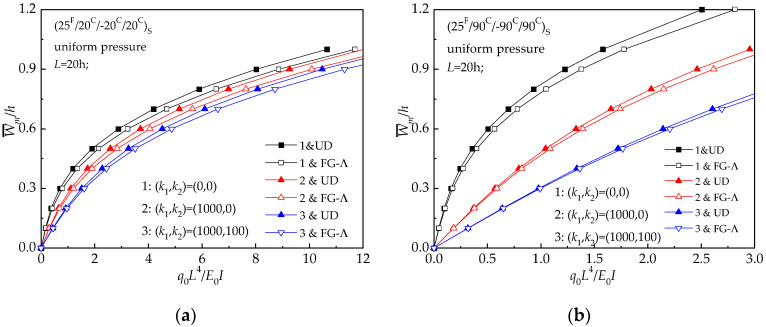
Load-deflection relationships of hybrid laminated beams with various foundation constants: (**a**) (25 ^F^/20 ^C^/−20 ^C^/20 ^C^) _S_; (**b**) (25 ^F^/90 ^C^/−90 ^C^/90 ^C^) _S._

Figure 14 plots the effect of the foundation stiffnesses on the bending characteristics of UD and FG-Λ with reference environmental conditions and *L* = 20 *h*. The predicted load-deflection curves are given for (*k*_1_, *k*_2_) = (0, 0), (10^3^, 0), (10^3^, 10^2^). In Figure 14, it is evident that the dimensionless central deflection decreases by increasing the foundation stiffnesses. Meanwhile, the effect of reinforcement of foundation on (25 ^F^/90 ^C^/−90 ^C^/90 ^C^) _S_ laminated beam is more obvious than the other beams.

## 7. Conclusions

In this study, a model for free vibration, non-linear forced vibration, and bending analyses of hybrid laminated beams with NPR and PPR under various external conditions is proposed. The model developed accounts for the FG configurations and NPRs in hybrid laminated beams. Five types of volume fractions of CNTs are considered which include: UD, FG-Λ, FG-V, and FG-X. For a configuration of (25 ^F^/20 ^C^/−20 ^C^/20 ^C^) _S_ and (25 ^F^/90 ^C^/−90 ^C^/90 ^C^) _S_, the out-of-plane Poisson’s ratios exhibit NPR and PPR, respectively. The solutions for the static and dynamic responses are derived by using a two-step perturbation approach by using a fourth-order Runge–Kutta method. The theoretical model predicts reliable values of frequencies and deflections in comparison with the results available in the existing model in the literature. The influence of the temperature variations and elastic foundation on the static and dynamic behavior of hybrid laminated beams is investigated in detail. 

Following comprehensive results are concluded:

- The distribution type of CNTs show considerable effect on the static and dynamic behavior of FRC/CNTRC plate with NPR or PPR. It is concluded that the FG-X beams have a lower frequency ratio. Moreover, for the non-linear bending and dynamic response, the FG-Λ beam showed better performance than that with UD under different external conditions.
- An increment in the temperature make the frequencies ratio and central defection considerably larger, whereas the increasing foundation stiffness will result in an opposite effect.
- The dynamic responses considering viscosity of foundation show obvious differences from those obtained with Pasternak foundation.
- The comparative studies reveal that the non-linear vibration of (25 ^F^/20 ^C^/−20 ^C^/20 ^C^) _S_ laminated beams are more sensitive to the temperature field, whereas, the non-linear bending in the (25 ^F^/20 ^C^/−20 ^C^/20 ^C^) _S_ laminated beams are less sensitive to the changes in temperature and foundation coefficient than those with (25 ^F^/90 ^C^/−90 ^C^/90 ^C^) _S_.

The results of the current work demonstrate that the NPR has a significant effect on the non-linear bending, free vibration, and forced-vibration characteristics of FRC/CNTRC hybrid laminated beams. Furthermore, the results show that the thermal-mechanical characteristics of FRC/CNTRC hybrid laminated beam can be remarkably improved by the determination of proper distribution of CNTs. For example, to have satisfactory performance in high temperature environments from hybrid laminated beams, beams with FG-X or FG-Λ arrangement can be preferred. Meanwhile, it is hoped that the current work will give an insight into the non-linear behavior of hybrid laminated beams with NPR and will be helpful for the further investigations.

## Figures and Tables

**Table 1:** Mechanical parameters with different temperature for each ply with CNTRC or FRC.

Volume Fraction	*T*	*E_11_*	*E_22_*	*G_12_*	*v*_12_	*α_11_*	*α_22_*
	(K)	(MPa)	(MPa)	(MPa)		(×10^−6^/K)	(×10^−5^/K)
*V_CN_* = 0.11	300 K	94,416.77	2203.74	822.28	0.3219	3.5830	5.3182
	400 K	92,708.58	1710.53	638.253	0.3219	4.2514	5.5640
	500 K	91,682.21	1217.32	454.222	0.3219	4.6123	5.8198
*V_CN_* = 0.14	300 K	120,384.60	2297.68	857.33	0.3169	3.5531	5.1582
	400 K	118,327.7	1783.46	665.46	0.3169	4.2269	5.3949
	500 K	117,144.40	1269.22	473.586	0.3169	4.5940	5.6414
*V_CN_* = 0.17	300 K	144,771.38	3493.88	1303.66	0.3120	3.5337	4.9979
	400 K	142,387.80	2711.95	1011.91	0.3120	4.2111	5.2255
	500 K	141,057.96	1930.01	720.144	0.3120	4.5821	5.4627
	300 K	140,670.00	5279.79	1731.78	0.2560	−0.2681	3.1446
*V_f_* = 0.6	400 K	140,482.00	4242.64	1380.01	0.2560	−0.3182	3.2665
	500 K	140,294.00	3130.95	1008.98	0.256	−0.3745	3.3885

**Table 2:** Distribution and volume fractions of CNT and fiber for hybrid beams.

	Types	UD	FG-X	FG-O	FG-Λ	FG-V	Symmetric Laminates in the Case Study
Each Ply							
Ply1		0.6	0.6	0.6	0.6	0.6	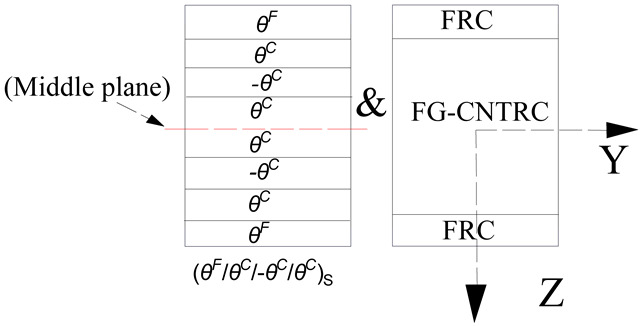
Ply2		0.14	0.17	0.11	0.11	0.17	
Ply3		0.14	0.14	0.14	0.11	0.17	
Ply4		0.14	0.11	0.17	0.14	0.14	
Ply5		0.14	0.11	0.17	0.14	0.14	
Ply6		0.14	0.14	0.14	0.17	0.11	
Ply7		0.14	0.17	0.11	0.17	0.11	
Ply8		0.6	0.6	0.6	0.6	0.6	

**Table 3:** EPRs ($v_{13}^{e}$) of hybrid laminated beams for various temperature conditions.

FG-	UD	FG-V	FG-Λ	FG-X	FG-O
(25^F^/20^C^/−20^C^/20^C^)_S_					
300K	−0.4566	−0.3972	−0.3972	−0.4213	−0.4213
400K	−0.5318	−0.5021	−0.5021	−0.5167	−0.5167
500K	−0.7263	−0.6583	−0.6583	−0.6743	−0.6743
(25^F^/90^C^/−90^C^/90^C^)_S_					
300K	0.3010	0.3016	0.3016	0.3017	0.3017
400K	0.3006	0.3012	0.3012	0.3013	0.3013
500K	0.3005	0.3009	0.3009	0.3010	0.3010

**Table 4:** Comparison of the fundamental frequencies $\tilde{\Omega }\;$ = Ω**(*L*^2^/*h*)$\sqrt{\rho_{0}/E_{0}}$ for (*θ^C^*/90^F^)_S_ hybrid beam with different values of the lamination angle of the CNTRC layer.

V_CN_		θ^C^		
		15	30	45
0.12	Fan & Wang [70]	5.27645	3.29216	2.79561
	Proposed method	5.28465	3.30550	2.81136
0.17	Fan & Wang [70]	6.66920	4.09226	3.42397
	Proposed method	6.67556	4.10290	3.43674
0.28	Fan & Wang [70]	7.12107	4.29687	3.57689
	Proposed method	7.12693	4.30689	3.58898

**Table 5:** Natural frequencies ${\tilde{\Omega }}_{i}\;$ = Ω*_i_*(*a^2^*/*h*)$\sqrt{\rho_{0}/E_{0}}$ for hybrid laminated beams in thermal environments (*L/h* = 5, *h* = 1.75 mm).

*T* (K)	Lay-up		${\tilde{\Omega }}_{1}$	${\tilde{\Omega }}_{2}$	${\tilde{\Omega }}_{3}$	${\tilde{\Omega }}_{4}$
300	(25 ^F^/20 ^C^/-20 ^C^/20 ^C^) _S_	UD	4.6911	13.6948	23.2622	32.9778
		FG-V&Λ	4.9235	14.3683	24.3888	34.5382
		FG-X	5.0219	14.3477	24.1203	34.0164
		FG-O	4.8071	14.5108	25.0357	35.7004
	(25 ^F^/90 ^C^/-90 ^C^/90 ^C^) _S_	UD	3.9290	12.4320	22.0451	31.9209
		FG-V&Λ	4.0516	12.8855	22.9136	33.2090
		FG-X	4.0952	12.8501	22.6652	32.7112
		FG-O	3.9949	12.9939	23.4395	34.2276
400	(25 ^F^/20 ^C^/-20 ^C^/20 ^C^) _S_	UD	3.5961	11.4037	19.6450	28.0129
		FG-V&Λ	3.9020	12.0972	20.7582	29.5337
		FG-X	3.9247	12.0007	20.4151	28.9379
		FG-O	3.7144	12.1788	21.3000	30.5349
	(25 ^F^/90 ^C^/-90 ^C^/90 ^C^) _S_	UD	3.3652	10.8818	19.3508	28.0456
		FG-V&Λ	3.4669	11.2728	20.1071	29.1715
		FG-X	3.4951	11.2254	19.8643	28.7019
		FG-O	3.4074	11.3733	20.5873	30.0945
500	(25 ^F^/20 ^C^/-20 ^C^/20 ^C^) _S_	UD	1.8258	8.3787	15.0130	21.7286
		FG-V&Λ	2.4974	9.2762	16.3236	23.4585
		FG-X	2.2548	8.9424	15.7088	22.5508
		FG-O	1.9929	9.1458	16.5824	24.0827
	(25 ^F^/90 ^C^/-90 ^C^/90 ^C^) _S_	UD	2.7546	9.1202	16.2644	23.5940
		FG-V&Λ	2.8368	9.4451	16.8979	24.5401
		FG-X	2.8478	9.3870	16.6674	24.1110
		FG-O	2.7746	9.5311	17.3136	25.3358

**Table 6:** Natural frequencies ${\tilde{\Omega }}_{i}\;$ = Ω*_i_*(*L^2^*/*h*)$\sqrt{\rho_{0}/E_{0}}$ for hybrid laminated beams resting on elastic foundations (*L/h* = 20, *h*=1.75 mm).

(*k*_1_, *k*_2_)	Lay-Up		${\tilde{\Omega }}_{1}$	${\tilde{\Omega }}_{2}$	${\tilde{\Omega }}_{3}$	${\tilde{\Omega }}_{4}$
(0, 0)	(25 ^F^/20 ^C^/-20 ^C^/20 ^C^) _S_	UD	5.5174	21.2692	45.2455	75.0582
		FG-V&Λ	5.7912	22.3244	47.4888	78.7764
		FG-X	6.0167	23.0823	48.7798	80.3508
		FG-O	5.5054	21.3661	45.8826	76.9132
	(25 ^F^/90 ^C^/-90 ^C^/90 ^C^) _S_	UD	4.5382	17.0453	37.0174	62.8646
		FG-V&Λ	4.4797	17.5337	38.1210	64.8256
		FG-X	4.5665	17.8376	38.6771	65.5239
		FG-O	4.3585	17.1122	37.3777	63.9190
(10^3^, 0)	(25 ^F^/20 ^C^/-20 ^C^/20 ^C^) _S_	UD	10.3970	23.0126	46.0837	75.5615
		FG-V&Λ	10.5449	23.9911	48.2880	79.2561
		FG-X	10.6704	24.6981	49.5584	80.8215
		FG-O	10.3907	23.1020	46.7089	77.4038
	(25 ^F^/90 ^C^/-90 ^C^/90 ^C^) _S_	UD	9.8311	19.1760	38.0361	63.4629
		FG-V&Λ	9.8855	19.6114	39.1109	65.4059
		FG-X	9.9251	19.8836	39.6435	66.0983
		FG-O	9.8312	19.2354	38.3866	64.5070
(10^3^, 10^2^)	(25 ^F^/20 ^C^/-20 ^C^/20 ^C^) _S_	UD	13.5920	28.8850	52.9492	81.062
		FG-V&Λ	13.7054	29.6705	54.8785	86.4789
		FG-X	13.8022	30.2454	56.0016	87.9216
		FG-O	13.5871	28.9558	53.4912	84.7756
	(25 ^F^/90 ^C^/-90 ^C^/90 ^C^) _S_	UD	13.1641	25.9308	46.1073	72.2569
		FG-V&Λ	13.2048	26.2543	46.9974	73.9677
		FG-X	13.2344	26.4583	47.4424	74.5835
		FG-O	13.1642	25.9744	46.3947	73.1687

## Appendix A

In Equations (9) and (10)

(A1) $$\begin{array}{c} S_{11}=\frac{4}{3h^{2}}\left(\frac{{\bar{B}}_{11}{\bar{E}}_{11}}{{\bar{A}}_{11}}-{\bar{F}}_{11}\right),\;S_{12}={\bar{D}}_{11}-\frac{4{\bar{F}}_{11}}{3h^{2}}+\frac{{\bar{B}}_{11}}{{\bar{A}}_{11}}\left(\frac{4}{3h^{2}}{\bar{E}}_{11}-{\bar{B}}_{11}\right),\\ S_{21}=\frac{4}{3h^{2}}\left[\frac{4}{3h^{2}}{\bar{H}}_{11}+\frac{{\bar{E}}_{11}}{{\bar{A}}_{11}}\left({\bar{B}}_{11}-\frac{4}{3h^{2}}{\bar{E}}_{11}\right)-{\bar{F}}_{11}\right],\\ S_{22}={\bar{D}}_{11}-\frac{4{\bar{F}}_{11}}{3h^{2}}+\frac{4}{3h^{2}}\left(\frac{4{\bar{H}}_{11}}{3h^{2}}-{\bar{F}}_{11}\right)-\frac{1}{{\bar{A}}_{11}}{\left({\bar{B}}_{11}-\frac{4{\bar{E}}_{11}}{3h^{2}}\right)}^{2},\\ S_{23}={\bar{A}}_{55}-\frac{4{\bar{D}}_{55}}{h^{2}}-\frac{4}{h^{2}}\left({\bar{D}}_{55}-\frac{4{\bar{F}}_{55}}{h^{2}}\right),\;S_{26}=\frac{1}{{\bar{A}}_{11}}\left({\bar{B}}_{11}-\frac{4{\bar{E}}_{11}}{3h^{2}}\right), \end{array}$$

(A2) $$({\bar{A}}_{11},\;{\bar{B}}_{11},\;{\bar{D}}_{11},\;{\bar{E}}_{11},\;{\bar{F}}_{11},\;{\bar{H}}_{11})=\sum_{k=1}^{N}b\int_{h_{k-1}}^{h_{k}}{\left({\tilde{Q}}_{11}\right)}_{k}(1,Z,Z^{2},Z^{3},Z^{4},Z^{6})dZ$$

(A3) $$({\bar{A}}_{55},{\bar{D}}_{55},{\bar{F}}_{55})=\sum_{k=1}^{N}b\int_{h_{k-1}}^{h_{k}}({\tilde{Q}}_{55}{)}_{k}(1,Z^{2},Z^{4})dZ$$

in which the detailed definition of ${\tilde{Q}}_{11}$ and ${\tilde{Q}}_{55}$ are found in [73]

(A4) $${\tilde{Q}}_{11}={\bar{Q}}_{11}+\frac{{\left({\bar{Q}}_{16}\right)}^{2}{\bar{Q}}_{22}-2{\bar{Q}}_{12}{\bar{Q}}_{16}{\bar{Q}}_{26}+{\left({\bar{Q}}_{12}\right)}^{2}{\bar{Q}}_{66}}{{\left({\bar{Q}}_{26}\right)}^{2}-{\bar{Q}}_{22}{\bar{Q}}_{66}},{\tilde{Q}}_{55}={\bar{Q}}_{55}-\frac{{\left({\bar{Q}}_{45}\right)}^{2}}{{\bar{Q}}_{44}},$$

and ${\left({\bar{Q}}_{ij}\right)}_{k}$ are the components of the transformed stiffness of kth lamina with orientation $\theta^{\left(k\right)}$, defined by

(A5a) $$\left[\begin{array}{l} {\bar{Q}}_{11}\\ {\bar{Q}}_{12}\\ {\bar{Q}}_{22}\\ {\bar{Q}}_{16}\\ {\bar{Q}}_{26}\\ {\bar{Q}}_{66} \end{array}\right]=\left[\begin{array}{cccc} c^{4} & 2c^{2}s^{2} & s^{4} & 4c^{2}s^{2}\\ c^{2}s^{2} & c^{4}+s^{4} & c^{2}s^{2} & -4c^{2}s^{2}\\ s^{4} & 2c^{2}s^{2} & c^{4} & 4c^{2}s^{2}\\ c^{3}s & cs^{3}-c^{3}s & -cs^{3} & -2cs(c^{2}-s^{2})\\ cs^{3} & c^{3}s-cs^{3} & -c^{3}s & 2cs(c^{2}-s^{2})\\ c^{2}s^{2} & -2c^{2}s^{2} & c^{2}s^{2} & {\left(c^{2}-s^{2}\right)}^{2} \end{array}\right]\;\left[\begin{array}{c} Q_{11}\\ Q_{12}\\ Q_{22}\\ Q_{66} \end{array}\right]$$

(A5b) $$\left[\begin{array}{c} {\bar{Q}}_{44}\\ {\bar{Q}}_{45}\\ {\bar{Q}}_{55} \end{array}\right]=\left[\begin{array}{cc} \begin{array}{c} c^{2}\\ -cs\\ s^{2} \end{array} & \begin{array}{c} s^{2}\\ cs\\ c^{2} \end{array} \end{array}\right]\left[\begin{array}{c} Q_{44}\\ Q_{55} \end{array}\right]$$

where

(A6) $$\begin{array}{c} Q_{11}=E_{11}{\left(1-v_{12}v_{21}\right)}^{-1},\;Q_{22}=E_{22}{\left(1-v_{12}v_{21}\right)}^{-1},\;Q_{12}=v_{21}E_{11}{\left(1-v_{12}v_{21}\right)}^{-1}\\ Q_{44}=G_{23},\;Q_{55}=G_{13},\;Q_{66}=G_{12} \end{array}$$

*E_11_*, *E_22_*, *v*_12_ etc… are summarized in Table 1.

In Equations (9) and (10), the coefficient *I_i_* (*i* = 1,2,3,4,5,7) can be calculated as:

(A7) $$(I_{1},I_{2},I_{3},I_{4},I_{5},I_{7})=\sum_{k=1}^{N}\rho_{k}\int_{h_{k-1}}^{h_{k}}\left(1,Z,Z^{2},Z^{3},Z^{4},Z^{6}\right)dZ$$

and

(A8) $$\begin{array}{c} {\bar{I}}_{2}=I_{2}-\frac{4I_{4}}{3h^{2}},\;{\bar{I}}_{3}=I_{3}-\frac{8}{3h^{2}}\left(I_{5}-\frac{2}{3h^{2}}I_{7}\right),\;{\tilde{I}}_{3}={\bar{I}}_{3}-\frac{{\bar{I}}_{2}{\bar{I}}_{2}}{I_{1}},\;{\bar{I}}_{5}=I_{5}-\frac{4I_{7}}{3h^{2}},\\ {\tilde{I}}_{5}={\bar{I}}_{5}-\frac{{\bar{I}}_{2}I_{4}}{I_{1}},\;{\hat{I}}_{5}={\tilde{I}}_{3}+\frac{4{\tilde{I}}_{5}}{3h^{2}},\;{\tilde{I}}_{7}=I_{7}-\frac{I_{4}I_{4}}{I_{1}},\;{\hat{I}}_{7}={\tilde{I}}_{5}+\frac{4{\tilde{I}}_{7}}{3h^{2}}, \end{array}$$

## Appendix B

In Equation (27)

(A9) $$\begin{array}{c} g_{30}=-\gamma_{17}+m^{2}\left(\gamma_{19}+\frac{\gamma_{18}\left(\gamma_{21}m^{2}-\gamma_{23}\right)}{\gamma_{22}m^{2}+\gamma_{23}}\right)+\frac{\gamma_{21}m^{4}}{\gamma_{22}m^{2}+\gamma_{23}}\left(\gamma_{29}+\frac{\gamma_{28}\left(\gamma_{21}m^{2}-\gamma_{23}\right)}{\gamma_{22}m^{2}+\gamma_{23}}\right),\\ g_{31}=m^{4}\left(\gamma_{11}-\frac{\gamma_{12}\left(\gamma_{21}m^{2}-\gamma_{23}\right)}{\gamma_{22}m^{2}+\gamma_{23}}\right)+(K_{1}+K_{2}m^{2})-\gamma_{T1}m^{2}\Delta T\\ +2\pi m\left(\gamma_{15}-\frac{\gamma_{14}\left(\gamma_{21}m^{2}-\gamma_{23}\right)}{\gamma_{22}m^{2}+\gamma_{23}}\right)\Phi +2\pi m^{2}\left(\gamma_{15}-\frac{\gamma_{14}\left(\gamma_{21}-\gamma_{23}\right)}{\gamma_{22}+\gamma_{23}}\right)\Phi +\frac{3\pi^{2}}{8}m^{2}\gamma_{13}\Phi^{2},\\ g_{32}=\{\begin{array}{c} 2\pi m^{3}\left(\gamma_{15}-\gamma_{14}\frac{\gamma_{21}m^{2}-\gamma_{23}}{\gamma_{22}m^{2}+\gamma_{23}}\right)-\frac{\pi^{2}}{4}3\gamma_{13}\Phi \;\mathrm{if}\;m=1\\ 2\pi m^{3}\left(\gamma_{15}-\gamma_{14}\frac{\gamma_{21}m^{2}-\gamma_{23}}{\gamma_{22}m^{2}+\gamma_{23}}\right)-\frac{\pi^{2}}{4}m^{2}\gamma_{13}\Phi \;\mathrm{if}\;m\neq 1 \end{array}\\ g_{33}=\frac{\pi^{2}m^{4}\gamma_{13}}{4}, \end{array}$$

in which

(A10) $$\Phi =\varepsilon A_{10}^{*}=\left(\frac{\lambda^{*}}{G_{10}}\right)-\frac{G_{20}}{G_{10}}{\left(\frac{\lambda^{*}}{G_{10}}\right)}^{2}-C_{33}{\left(\frac{\lambda^{*}}{G_{10}}\right)}^{3}+\cdots$$

where

(A11) $$\begin{array}{c} \lambda^{*}=\frac{4}{\pi }\left(\gamma_{T3}-\frac{\gamma_{12}(\gamma_{T3}-\gamma_{T6})}{\gamma_{22}+\gamma_{23}}\right)\Delta T,\\ G_{10}=\left(\gamma_{11}-\frac{\gamma_{12}\left(\gamma_{21}-\gamma_{23}\right)}{\gamma_{22}+\gamma_{23}}\right)+(K_{1}+K_{2})+\frac{2\pi \gamma_{14}(\gamma_{T3}-\gamma_{T6})\Delta T}{\gamma_{22}+\gamma_{23}},\\ G_{20}=2\pi \left(\gamma_{15}-\frac{\gamma_{14}\left(\gamma_{21}-\gamma_{23}\right)}{\gamma_{22}+\gamma_{23}}\right),\;G_{30}=\frac{\pi^{2}\gamma_{13}}{4},\;C_{33}=\frac{G_{30}}{G_{10}}-{\left(\frac{\sqrt{2}G_{20}}{G_{10}}\right)}^{2} \end{array}$$
